# Diffuse Bi-hemispheric Cortical Infarction Secondary to Cerebral Air Embolism After Elective Esophagogastroduodenoscopy: A Case Report and Review of Literature

**DOI:** 10.7759/cureus.36069

**Published:** 2023-03-13

**Authors:** Rahul Shah, Saumya Shah

**Affiliations:** 1 Neurology/Neuro Critical Care, Bakersfield Memorial Hospital, Bakersfield, USA; 2 Neurology, California Health Sciences University - College of Osteopathic Medicine, Bakersfield, USA

**Keywords:** deep sea diving, high mortality, patent foramen oval, cortical laminar necrosis, neuro-critical care, coma, venous gas embolism, hyperbaric oxygen thetapy, esophagogastroduodenoscopy (egd), cerebral air embolism

## Abstract

Cerebral air embolism (CAE) is a rare entity and is more often secondary to iatrogenic causes. We present a rare case of CAE post esophagogastroduodenoscopy (EGD) with a shower of emboli resulting in diffuse cortical infarction.

An 80-year-old man with gastroesophageal reflux disease underwent an elective EGD for esophageal dilatation. During the procedure, there was significant arterial and venous bleeding which subsided with treatment. After the procedure, the patient became comatose with the decerebrate extensor posturing to painful stimulation. Computed tomography without contrast revealed multiple foci of CAE. Diffusion-weighted magnetic resonance images performed at the same time showed numerous areas of acute ischemic lesions affecting primarily the Gray Matter, demonstrating both cortical micro air emboli and bi-hemispheric, global hypoperfusion leading to cortical ribboning pattern.

There have been less than 22 cases of CAE after EGD in the literature, most of which were middle cerebral arterial territory infarctions. Our clinical images represent one of the extremely rare cases showing a shower of emboli and global hypoperfusion-induced gray matter infarction due to CAE-causing brain herniation.

## Introduction

Cerebral air embolism (CAE) is an uncommon entity with high morbidity and mortality that can oftentimes be fatal. It is frequently observed secondary to iatrogenic causes - neurosurgical procedures, bronchoscopies, vascular surgeries, gastric procedures like esophagogastroduodenoscopy (EGD), and Endoscopic Retrograde Cholangiopancreatography (ERCP), central line placements, positive pressure ventilation, etc., and deep-sea diving [1,2]. Presentations range from headaches, and seizures - both focal and generalized, to focal neurological deficits, decreased consciousness, and coma. CAE after EGD is not commonly reported [3,4]. This is an unfortunate but extremely rare case of CAE post-EGD with a shower of emboli causing extensive, diffuse, bilateral cortical infarctions.

## Case presentation

**Figure 1:**
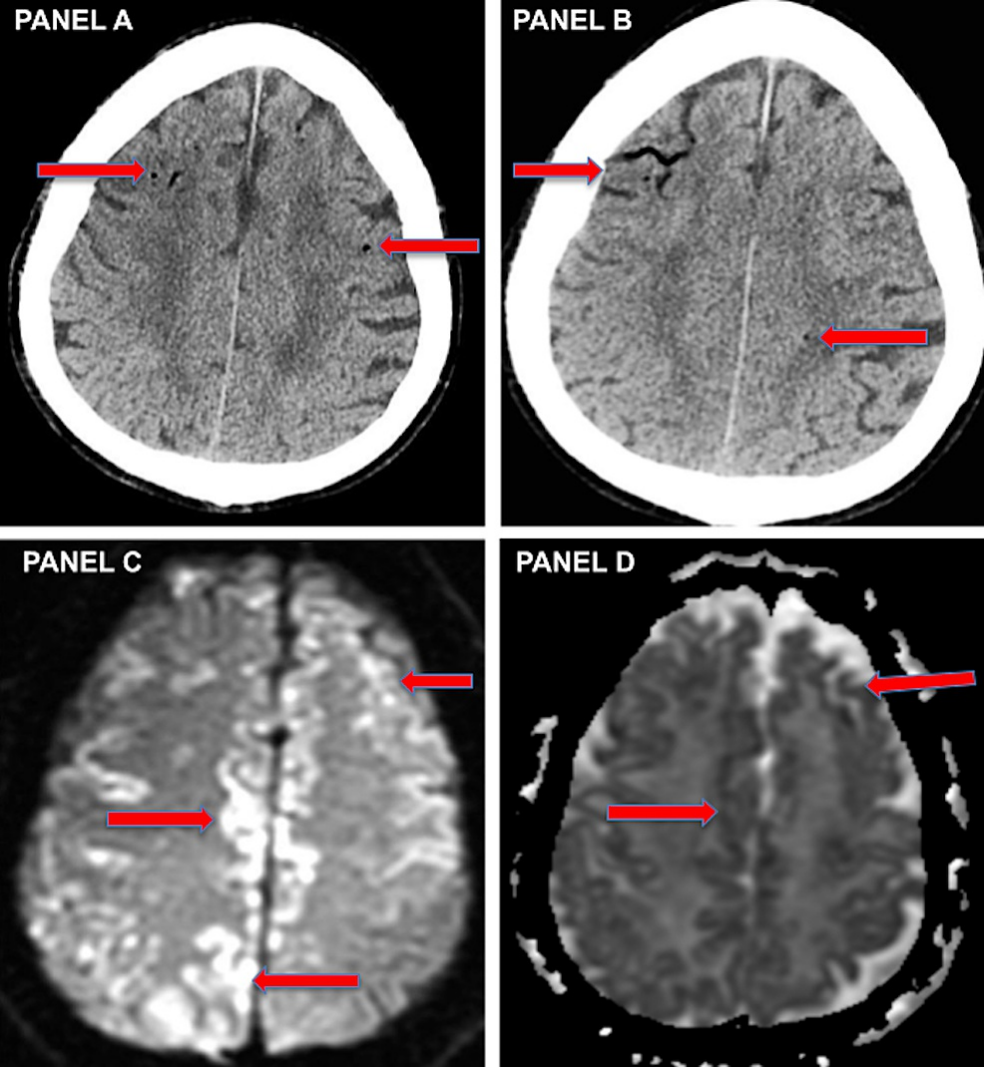
CT head non-contrast and MRI brain without contrast images (A,B) CT head non-contrast: axial cuts showing multiple foci of air in bilateral cerebral arteries in both hemispheres. (C, D) MRI brain DWI and ADC sequence axial cuts showing diffuse areas of restricted diffusion with bilateral gray matter involvement and cortical ribboning pattern.

An 80-year-old Caucasian man with gastroesophageal reflux disease underwent an elective EGD for esophageal dilatation. The patient was under monitored anesthesia care (MAC) anesthesia; an endoscope was passed and some gastric erythema, as well as a 12-mm distal esophageal stricture, was visualized. Stricture was biopsied and the balloon dilated through it to about 15 mm. Entire procedure lasted about five to seven minutes with no obvious immediate complications. No excessive bleeding or vascular issues were noted as per documentation. Status post EGD, the patient did not wake up from the anesthesia and was unresponsive. A stroke code was called, and the patient was transferred to the neurocritical care unit at Memorial Hermann hospital for a higher level of care. On admission, the patient’s neurological exam was significant for GCS of 4 with extensor posturing on painful stimuli. Computed tomography (CT) without contrast revealed multiple foci of air in multiple cerebral arteries in bilateral hemispheres suggesting diffuse CAE (Figures 1A, 1B). Subsequent CT angiogram (CTA) of the brain did not reveal any occlusions. Diffusion-weighted imaging (DWI) and apparent diffusion coefficient (ADC) imaging sequences on magnetic resonance imaging (MRI) performed thereafter revealed numerous areas of acute ischemic lesions affecting primarily the cortex. MRI findings were demonstrative of bi-hemispheric, global cerebral hypoperfusion leading to the cortical ribboning pattern (Figures 1C, 1D). CT chest showed pneumomediastinum. Considering the delay in presentation to our ICU, subsequent delay in diagnosis, and MRI findings already suggestive of extensive cerebral ischemia with extremely poor clinical exam, Hyperbaric Oxygen therapy was not offered. The patient showed no improvement in his overall neurological status over the next 48 hours. The family decided to transition the patient to comfort care and he was compassionately extubated two days later and died immediately thereafter.

## Discussion

This case represents an extremely rare and infrequent but oftentimes fatal complication of EGD and associated interventional procedures. We performed a search on PUBMED and Google Scholar with the keywords “EGD and air embolism,” “EGD and cerebral gas embolism,” and “EGD and cerebral air embolism” to look at the incidence of CAE after EGD. All studies including case reports and case series were included. We excluded cases and studies which involved associated surgical procedures. Cases with only ERCP as the preceding procedure were excluded as well since we wanted to focus solely on the incidence of CAE after EGD. Any duplicates were excluded. Based on our literature search, we found only 22 previously reported cases of CAE from EGD. Five patients reportedly received hyperbaric oxygen (HBO) therapy [4]. Of the total patients, only 12 survived, with two out of those 12 receiving HBO. A documented patent foramen ovale (PFO) or some form of arteriovenous (AV) shunt was seen in 12 patients.

Air embolism occurs when air inadvertently finds entry into the vascular space and can be venous or arterial. Both situations can be equally fatal with high rates of morbidity and mortality. The most common causes are iatrogenic, with procedures such as EGD, ERCP, interventional radiological procedures, positive pressure ventilation, cardiothoracic surgeries, and neurosurgical procedures being implicated amongst others. Pulmonary embolism is the most common presentation. CAE, as seen in our patient, is far less common. We have focused on CAE after GI procedures, more specifically, EGD in our discussion. 

Different theories regarding how air enters the arterial space after EGD to cause CAE have been postulated. Direct air entry through exposed blood vessels secondary to therapeutic endoscopic procedures involving biopsies or that result in a direct vessel or mucosal injury is one mechanism. Air may enter directly through exposed vessels within gastric ulcers or gastric tumors. The higher-pressure gradients secondary to the insufflation of air during the procedure are further hypothesized to facilitate air entry [5,6]. Translocation of air from the venous circulation to the systemic circulation can be secondary to intracardiac shunts like PFO. Intrapulmonary shunting through arteriovenous malformations or large volumes of air overwhelming the pulmonary vasculature’s ability to filter the air is another mechanism by which air transfers over from the venous circulation to the arterial circulation.

Our patient did not have a documented Echocardiogram with a shunt study, so it was unknown if he did indeed have a PFO. Considering the extremely low incidence of CAE after EGD and the fact that about 25% of the general population has a PFO precludes the need to check for PFO in every patient prior to routine EGD [1,5,7]. There is no clear consensus on the efficacy and benefit of peri-procedural monitoring for venous air embolization (VAE). Transesophageal echocardiogram (TEE) has one of the highest sensitivities but is invasive with its own set of risks. Precordial Doppler ultrasound is an extremely sensitive and non-invasive test, and more and more institutes are incorporating its use in high-risk cases. Transcranial Doppler (TCD) is highly sensitive to diagnosing PFOs and is being more frequently used for screening for high-risk surgeries [8]. Despite all these useful modalities, the earliest and most sensitive method of identifying possible VAE during procedures is clinical suspicion. Any decrease in the level of consciousness, lethargy, delay in regaining consciousness after extubation and reversal of sedation, unexplained hypoxia, sudden onset focal neurological deficits, or sometimes, direct visualization of air entering the vascular space should all raise enough suspicion to abort the procedure, decompress the stomach to prevent further air entry, and proceed with diagnostic testing to confirm the diagnosis. CT head and CTA may show intraparenchymal or intravascular gas, but they are strongly dependent on the volume of gas within the vasculature as well as duration since ictus. Although highly specific, they are often negative [8,9].

Recent studies have shown that early identification and early initiation of HBO therapy result in improved outcomes in CAE patients. HBO is believed to help by decreasing the size of gas emboli and providing higher oxygen concentrations in arterial blood which may benefit the ischemic tissue [1,2,4,6]. Initiating HBO therapy more than six hours after insult, as well as early and significant ischemic changes seen on CT/MRI before starting therapy were both considered strong predictors of poor neurological and clinical outcomes [1,2,4]. Unfortunately, a significant portion of patients is under sedation for the procedure, often resulting in delays in clinical diagnosis and initiating treatment, further resulting in poor outcomes [6,8].

Our patient not receiving HBO was down to multiple factors. He arrived at our institute more than 10 hours after the event, his neurological exam was extremely poor, and his MRI already showed extensive bilateral ischemic changes indicating global diffuse cerebral ischemia, all suggestive of an extremely poor prognosis.

## Conclusions

CAE post EGD resulting in global cerebral bi-hemispheric ischemia as seen in this patient is highly uncommon. Visible foci of air in multiple vascular territories as seen in our patient's CT scan are even more uncommon. CTA, although highly specific, is commonly negative and is dependent on the volume of air present in vasculature. Although HBO has been shown to improve outcomes, early initiation of treatment is paramount. Time to treatment is more than six hours, and early CT/MRI evidence of ischemic changes suggests poor outcomes. Whether preoperative surveillance for PFOs or perioperative surveillance for venous air emboli in high-risk cases presents a benefit in prevention is unclear, although the high fatality and poor outcomes in cases these cases may suggest value in incorporating these surveillance modalities for selective procedures.
